# Impact of device design on the electronic and optoelectronic properties of integrated Ru-terpyridine complexes

**DOI:** 10.3762/bjnano.13.16

**Published:** 2022-02-15

**Authors:** Max Mennicken, Sophia Katharina Peter, Corinna Kaulen, Ulrich Simon, Silvia Karthäuser

**Affiliations:** 1 Peter Grünberg Institut (PGI-7) and JARA-FIT, Forschungszentrum Jülich GmbH, 52425 Jülich, Germany; 2 RWTH Aachen University, 52062 Aachen, Germany; 3 Institute of Inorganic Chemistry and JARA-FIT, RWTH Aachen University, 52074 Aachen, Germany; 4 Faculty of Medical Engineering and Applied Mathematics, FH Aachen, University of Applied Science, 52428 Jülich, Germany

**Keywords:** conductance switching, gold nanoparticles, nanoelectronic devices, optical addressing, Ru-terpyridine wires

## Abstract

The performance of nanoelectronic and molecular electronic devices relies strongly on the employed functional units and their addressability, which is often a matter of appropriate interfaces and device design. Here, we compare two promising designs to build solid-state electronic devices utilizing the same functional unit. Optically addressable Ru-terpyridine complexes were incorporated in supramolecular wires or employed as ligands of gold nanoparticles and contacted by nanoelectrodes. The resulting small-area nanodevices were thoroughly electrically characterized as a function of temperature and light exposure. Differences in the resulting device conductance could be attributed to the device design and the respective transport mechanism, that is, thermally activated hopping conduction in the case of Ru-terpyridine wire devices or sequential tunneling in nanoparticle-based devices. Furthermore, the conductance switching of nanoparticle-based devices upon 530 nm irradiation was attributed to plasmon-induced metal-to-ligand charge transfer in the Ru-terpyridine complexes used as switching ligands. Finally, our results reveal a superior device performance of nanoparticle-based devices compared to molecular wire devices based on Ru-terpyridine complexes as functional units.

## Introduction

Discernible properties of nanoelectronic and molecular devices are directly influenced by the molecular structure of the constituting molecular systems, the intermolecular and interfacial interactions and the design of the device. Molecular engineering is required to design and assemble molecules or supramolecular systems with specific functions and to ensure the device performance. Favorable molecular systems are capable of performing electronic operations such as data storage, rectification, sensing or switching. In this regard metallo-supramolecular wires are promising candidates as electronic and luminochromic materials, which are also useful in a variety of other applications [1–3]. They are available with different transition metal centers and numerous chelating ligands. The ligands may be designed to promote the wire growth, to include additional functionalities or to form bridges to biomolecules [4–5]. Common ligands, particularly suitable for the metallo-supramolecular wire growth and exhibiting high binding constants to a broad range of transition metal ions, are often based on 2,2′:6′,2″-terpyridine (TP) motives [5–7]. TP complexes, themselves or as hybrid materials with (semi)conducting species, are redox-active and, thus, enable applications in nanoelectronics and catalysis [8–10]. Among the suitable transition metal centers, Ru is highly attractive since Ru(TP)_2_-complexes show intense metal-to-ligand charge transfer absorption bands and a relatively long lifetime of the triplet state as well as voltage-driven molecular switching in solid-state molecular junctions [11–15]. In addition, Ru(TP)_2_-complexes and the corresponding supramolecular wires exhibit a rod-like structure, which makes them superior candidates for charge transport studies and functional nanodevices [16–18].

Using TP-based ligands and a reactive Ru precursor, we recently succeeded to establish a room-temperature method to grow Ru-TP supramolecular wires by sequential reaction [19]. The stepwise wire growth was verified by infrared reflection absorption spectroscopy (IRRAS) and surface-enhanced Raman spectroscopy in combination with density functional theory calculations, as well as variable angle spectroscopic ellipsometry. Based on this wire formation protocol the on-chip preparation of Ru(TP)_2_-complex wires to bridge a nanometer-sized gap between two electrodes becomes accessible. The challenge is to integrate these redox-active Ru(TP)_2_-complex wires reliably into a device geometry such that the envisaged device properties are established. Here, our approach is to employ nanodevices equipped with nanoelectrodes separated by gaps of 8 to 20 nm. They are fabricated by electron beam lithography (EBL) in a lift-off process while using a self-aligned Al_2_O_3_ hard mask to define the nanogap size [20–21]. The resulting nanoelectrode pairs are used for the on-chip preparation of Ru(TP)_2_-complex wires according to the wire-growth protocol developed recently and, thus, to assemble Ru(TP)_2_-complex wire nanodevices [19].

Additionally, we will use our already successfully applied approach to fabricate nanodevices from ligand-stabilized gold nanoparticles (AuNPs) immobilized between nanoelectrodes [21–24]. Very recently, we have assembled nanodevices based on single AuNPs with a diameter of 14 nm and stabilized by twofold positively charged bis{4′-[4-(mercaptophenyl)-2,2′;6′,2″-terpyridine]}ruthenium complexes (Ru(MPTP)_2_). A thorough electronic characterization of these single-AuNP devices indicates that the redox behavior of the Ru(MPTP)_2_-complexes is preserved in the device geometry [15]. Here, we use nanodevices equipped with gaps between the nanoelectrodes in the range of 20 to 50 nm so that a countable number of Ru(MPTP)_2_–AuNP building blocks is needed to bridge the nanogaps. The thus formed Ru(MPTP)_2_–AuNP devices are easier to fabricate and are assumed to be of higher technological relevance.

In this study, the focus of our attention lies on the relation between device design and device performance, that is, which device design is suited best to guarantee the functionality of the constituting building blocks. For this purpose, the transport properties of the redox-active Ru(TP)_2_-complexes are studied under electrical or optical triggering in Ru(TP)_2_-complex wire devices and in Ru(MPTP)_2_–AuNP devices. Both devices are based on the same nanoelectrode design and are composed of analogous molecular building blocks. However, the nanogaps are bridged either directly by long Ru(TP)_2_-complex wires or with AuNPs functionalized with TP-based Ru complexes. We will show that it is not only a challenge to design and assemble a molecular device, but to address the constituting elements so that the device physics is mainly determined by the molecular properties.

## Experimental

### Chemical synthesis

4’-Mercaptophenyl-2,2′:6′,2″-terpyridine (MPTP), 4′-[4-(acetylthio)phenyl]-2,2′:6′,2″-terpyridine (MPTP-SAc), 1,4-bis(2,2′:6′,2″-terpyridine-4-yl)benzene (BTP), the complex RuCl_2_(DMSO)MPTP-SAc and the precursor [Ru(acetone)_6_](PF_6_)_3_ (Ru-PF_6_) were prepared according to procedures previously described [15,19,25–27]. The analysis of the prepared substances, including ^1^H NMR, UV–vis spectroscopy and mass spectrosmetry, display a high purity [15]. The molecular formulas of the chemical compounds are given in Supporting Information File 1, Figure S1.

Ru complex-functionalized AuNPs were synthesized by ligand complexation of MPTP–AuNPs with RuCl_2_(DMSO)MPTP-SAc in acetic acid solution using the protocol given recently [15]. The thorough characterization revealed spherical nanoparticles, Ru(MPTP)(MPTP-SAc)–AuNPs, with an average size of 12.9 ± 1.6 nm (Supporting Information File 1, Figure S2).

### Ru(TP)_2_-complex wire growth

For the tracking of the Ru(TP)_2_-complex wire formation via XPS measurements mica substrates of 8 × 4 mm^2^ covered by a 200 nm Au(111) layer were used. In a first step the substrates were treated with MPTP solution (1 mM in ethanol) over 24 h at RT. The Ru(TP)_2_ wire growth was performed by (repeatedly) incubating the samples in Ru-PF_6_ dissolved in ethanol (1 mM) over 5 min and subsequently in BTP solution (1 mM in chloroform) over 20 min at 60 °C. After each step, the samples were rinsed three times in either ethanol or chloroform.

### Nanodevice fabrication

The fabrication of nanoelectrode samples, each equipped with twelve nanoelectrode pairs (consisting of an AuPd and a Pt electrode with a nanometer-sized gap in between), was performed according to a recently described process using electron beam lithography and lift-off [21]. These nanoelectrode samples with gap sizes of 8 to 20 nm between the electrodes were used in order to fabricate Ru(TP)_2_-complex wire devices. According to the Ru-complex wire growth procedure described above, the samples were treated first with MPTP solution followed by alternately employing Ru-PF_6_ and BTP solution.

Nanoelectrode samples with 20 to 50 nm gaps were used to assemble devices based on multiple Ru(MPTP)_2_–AuNP building blocks. For this purpose, a droplet of the Ru(MPTP)(MPTP-SAc)–AuNP dispersion was deposited onto the nanoelectrode structure such that an almost complete nanoparticle layer was formed. In a next step, the acetyl-protected thiol groups of MPTP-SAc were hydrolyzed with concentrated ammonium solution yielding mainly Ru(MPTP)_2_–AuNP devices, followed by a washing procedure [15]. As usual, all nanoelectrode samples have been electrically characterized before use to rule out material artifacts. Only nanogaps with a resistance above 10 TΩ were employed for further experiments. Representative SEM images of empty nanogaps, Ru(TP)_2_-complex wire devices and Ru(MPTP)_2_–AuNP devices are given in Supporting Information File 1, Figure S3.

### Instrumentation

X-ray photoelectron spectroscopy (XPS) measurements were conducted with a PHI5000 VersaProbe II using monochromatic Al Kα radiation (1.486 keV). Survey scans (187.5 eV pass energy, 0.8 eV/step) and core level spectra (23.5 eV pass energy, 0.1 eV/step) of the elements C 1s, N 1s, O 1s, S 1s, Ru 3d, and Au 4f were recorded. Data analysis was conducted using CasaXPS (Casa Software, Ltd.) after subtraction of a Shirley background. The binding energies (BE) were calibrated to give the signal for metallic gold Au 4f_7/2_ at 84.0 eV.

Scanning electron microscopy (SEM) was performed with a Hitachi SU8000 apparatus using an acceleration voltage of 5 kV.

The electronic characterization of the Ru(TP)_2_-complex wire devices as well as the Ru(MPTP)_2_–AuNP devices was conducted with a Keithley 6430 sub-femtoampere remote source meter as described recently [21]. Current-voltage (*I*/*U*) characteristics were taken in the temperature range from 300 to 360 K at 1 bar pressure under helium atmosphere in an optical continuous flow cryostat (Oxford Optistat CF). Optical switching experiments were performed by applying light with the discrete wavelength in the range of 440 to 540 nm using a Hg arc lamp and subsequent wavelength selection with monochromator (MS257 Oriel). The radiant power was 90 nW/cm^2^ and the optical setup was coupled via a fiber-cable to the optical cryostat.

## Results and Discussion

### Ru(TP)_2_-complex wire growth

**Figure 1:**
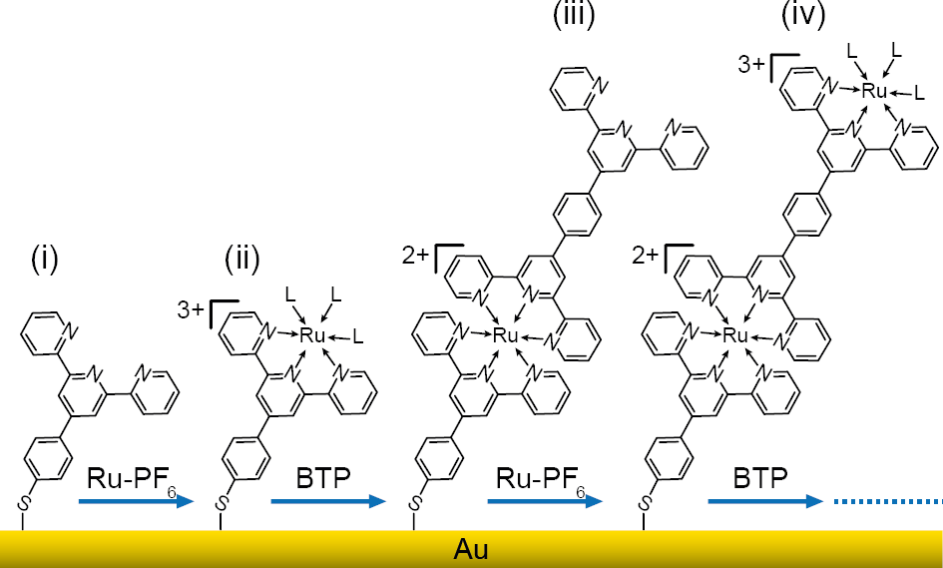
Stepwise Ru(TP)_2_-complex wire growth by alternate addition of the Ru-PF_6_ precursor in ethanol and BTP solution, (i)–(iv) see text above; L = ligand (acetone or ethoxide anion).

Ru(TP)_2_-complex wires were grown between nanoelectrodes fabricated via electron beam lithography according to the recently developed routine that enables the assembly of the molecular building blocks on solid surfaces at room-temperature under mild conditions and, thus, represents an on-chip preparation method [19]. In parallel, the first steps of the consecutive wire growth on Au substrates were monitored by XPS measurements. The step-by-step formation of the Ru(TP)_2_-complex wires was conducted starting with the assembly of MPTP on Au substrates, corresponding to samples (i) (Figure 1). Then the substrates were incubated in a Ru-PF_6_ solution, leading to the formation of the Ru-MPTP half-facial complexes on the surface, representing samples (ii). Subsequently incubating the substrates in BTP solution leads to MPTP-Ru-BTP wires, corresponding to samples (iii). The wire growth was completed by incubation of samples (iii) in Ru-PF_6_ solution for a second time, thereby forming samples (iv) (Figure 1).

Recently, we showed by evaluation of the IRRAS and Raman spectra of the individual wire growth steps (i)–(iii) and comparison of these spectra to the spectra of bulk model substances that the formation of the Ru(TP)_2_ complexes was largely successful [19]. However, a detailed investigation of the oxidation state of the Ru ions bound to the respective molecular compounds is not feasible by these methods. In contrast, XPS BE highly depend on the oxidation state and/or the chemical composition and are, thus, a suitable means to reveal details of the Ru(TP)_2_-complex wire growth process. Therefore, the core level spectra of C 1s, Ru 3d, and O 1s have been recorded after each growth step and the obtained values are given in Table 1 (see Supporting Information File 1 for in depth analysis, section 4, and XPS core level spectra, Figure S4). Overall, the result of the detailed XPS analysis is that the wire-terminating group related to the respective growth step can be readily identified by the corresponding BE for the C 1s, Ru 3d, and O 1s core levels. Growth steps (i) and (iii) characterized by a terminal TP group revealing a low intensity C 1s peak at 286.5 eV (C–N), which shifts to 286.2 eV (C–O) for growth steps (ii) and (iv) indicating a termination by the Ru(TP)(L)_3_-complex. The related Ru 3d_5/2_ peak at 282.1 eV is identified as Ru^3+^ bound to ethoxide ions for (ii) and (iv) while the Ru peak at 281.5 eV found for the MPTP-Ru-BTP wires (iii) clearly indicates Ru^2+^ corresponding to Ru(TP)_2_-complexes [28]. Likewise, the O 1s peak assignment reveals distinctly the alternation of the terminal groups during the Ru(TP)_2_-complex wire growth. The main O 1s peak in the spectra of sample (ii) and sample (iv) denotes the termination of the wire by the Ru(TP)(L)_3_-complex in accordance with the C 1s and Ru 3d_5/2_ spectra, while the O 1s peak for growth steps (i) and (iii) points to the sporadic adsorption of ethanol to the terminal TP groups [29–30]. Thus, the XP spectra prove, in addition to earlier published results based on other spectroscopic methods, that the desired wire growth has been successfully conducted and reveal the oxidation state of Ru during complex wire growth [19].

**Table 1:** Binding energies (in eV) of C 1s, Ru 3d_5/2_, and O 1s core levels given for the consecutive wire growth steps shown in Figure 1.^a^

		C 1s			Ru 3d_5/2_		O 1s		
		C–C	C–O	C–N	Ru^2+^	Ru^3+^	C–O^−^	C–OH	C–OH···N

(i)	MPTP	**285.5**	—	286.7	—	—	—	**532.9**	534.2
(ii)	MPTP-Ru	**285.0**	286.2	—	—	**282.1**	**531.8**	533.0	—
(iii)	MPTP-Ru-BTP	**284.8**	—	286.5	**281.5**	282.3	531.7	**532.5**	534.6
(iv)	MPTP-Ru-BTP-Ru	**284.9**	286.2	—	281.3	**282.2**	**531.4**	532.6	—

^a^BE of the main peaks are given in bold.

### Electrical properties of Ru(TP)_2_-complex devices

**Figure 2:**
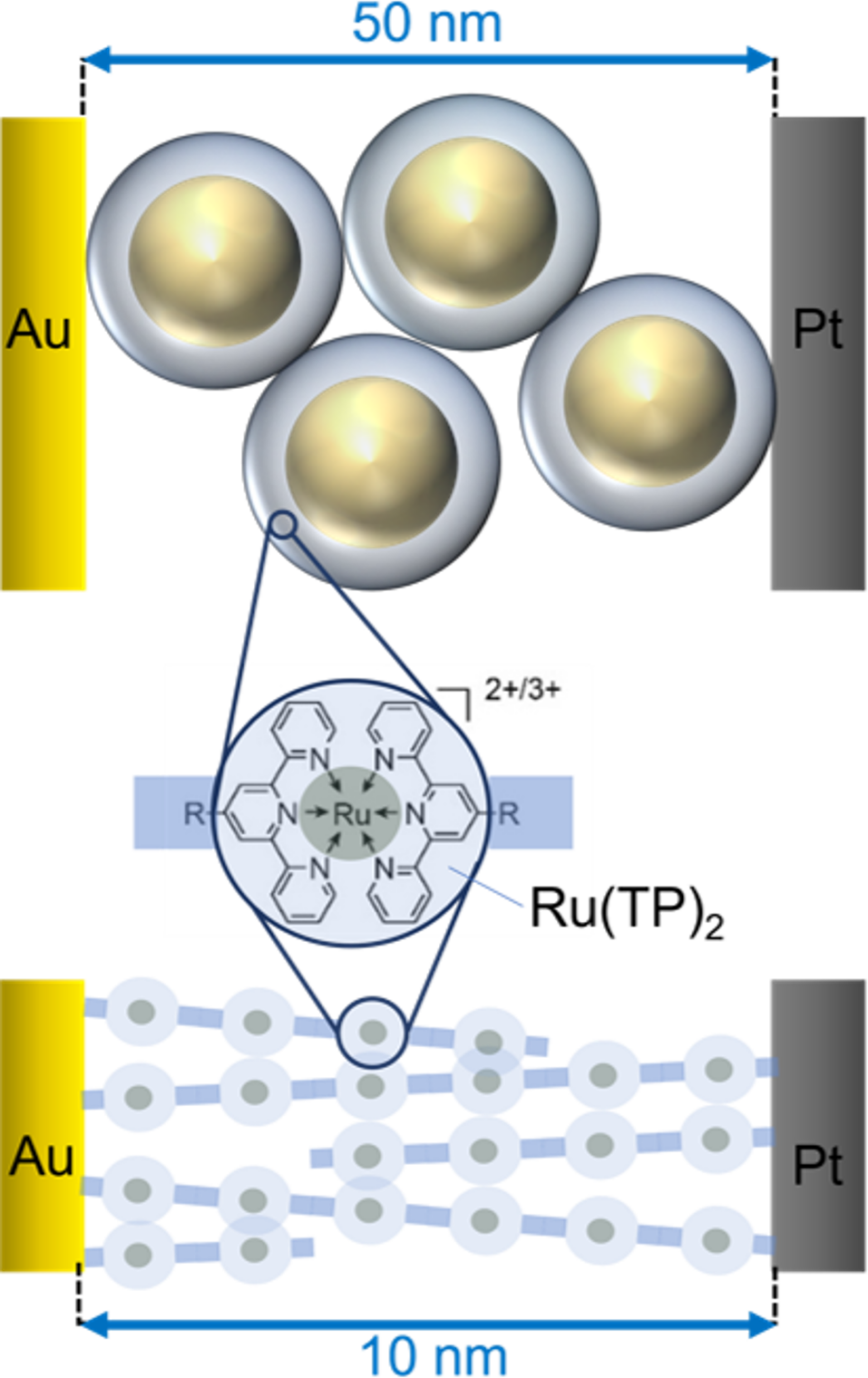
Schematic illustration of Ru(MPTP)_2_–AuNP (upper part) and Ru(TP)_2_-complex wire devices (lower left part) based on the same functional molecular unit, Ru(TP)_2_ (not drawn to scale).

Here, we study the relation between device design and device performance and compare the transport and optical switching properties of functional Ru(TP)_2_-complexes in supramolecular wire devices and in AuNP nanodevices. Both devices are based on the same nanoelectrode design and on the identical redox-active Ru(TP)_2_-complexes (Figure 2). Furthermore, these devices will be compared to single-Ru(MPTP)_2_–AuNP devices described recently [15].

**Figure 3:**
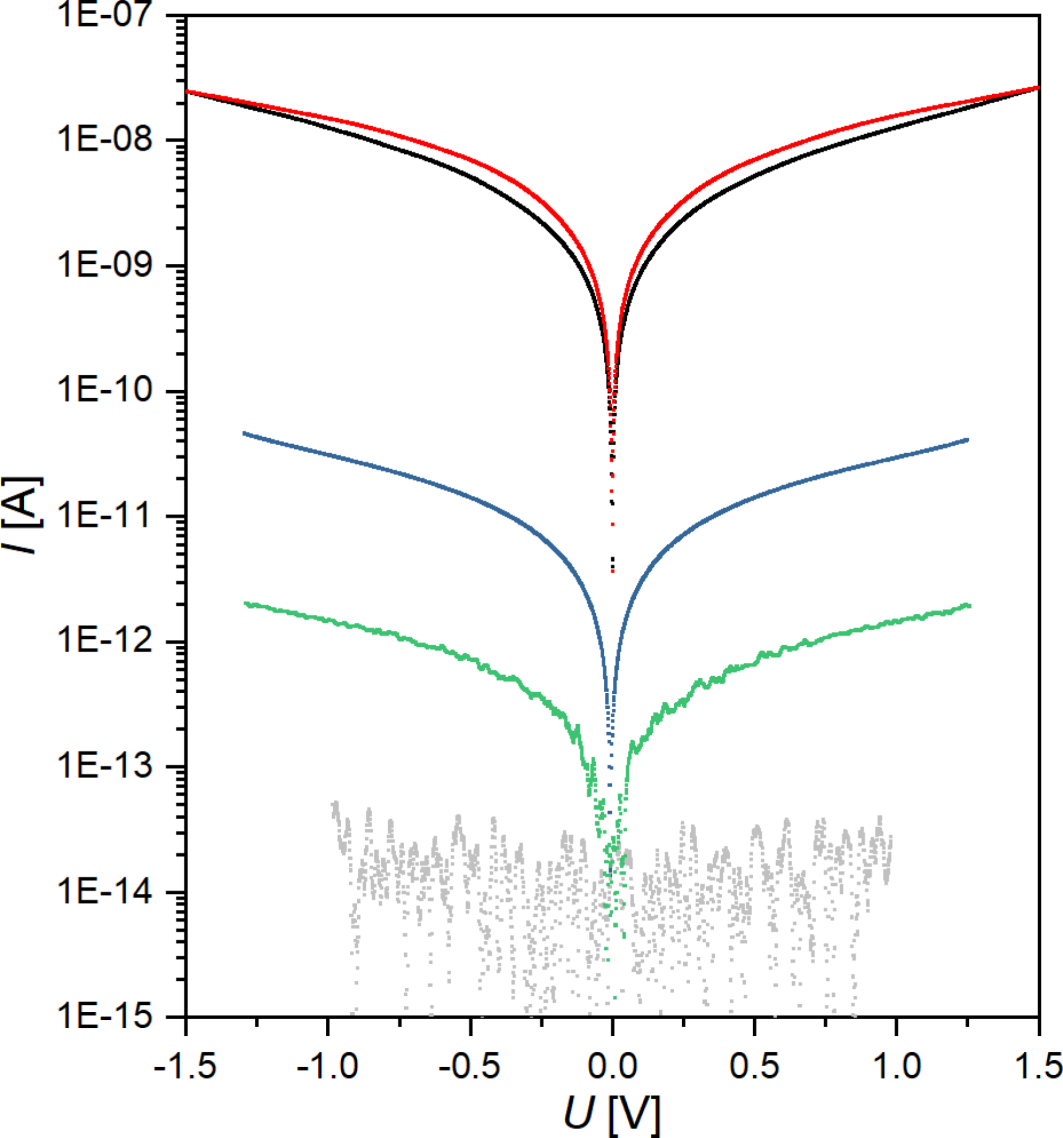
Log current vs voltage curves of Ru(TP)_2_-complex wire devices (green), multiple-Ru(MPTP)_2_–AuNP devices (blue), single-Ru(MPTP)_2_–AuNP devices (red/black, data taken from [15]), and empty nanogaps representing the noise level (grey).

Ru(TP)_2_-complex wire devices have been fabricated using nanoelectrodes with a separation of 8 to 20 nm and the above described growth process. The electrical behavior of the thus formed solid-state junctions was measured by cyclic current vs voltage (*I*/*U*) sweeps in the voltage range −1.3 V < *U* < +1.3 V. The resulting conductance values (at 1 V) are typically found in the range from 0.74 to 1.49 pS with a median of 1.35 pS (Supporting Information File 1, Figure S5) and no dependence on the nanoelectrode gap size exceeding the fluctuations between different devices is detected. It should be noted that reference measurements on empty nanoelectrode gaps or nanoelectrode pairs only treated repeatedly with TP solution and ethanol show conductance values around 0.01 pS, corresponding to the noise level. In Figure 3 the current vs voltage graph of a typical Ru(TP)_2_-complex wire device (green) is given, exhibiting a low conductance value characteristic for long supramolecular wires. These nanowire devices are compared to well-accessible devices based on a few Ru(MPTP)_2_–AuNP building blocks with a diameter of 12.9 ± 1.6 nm immobilized between nanoelectrode pairs with gap sizes of 20 to 50 nm. Here, the redox-active Ru(MPTP)_2_-complexes form the ligand shell of the AuNPs. In these devices two to four Ru(MPTP)_2_–AuNP building blocks are needed to bridge the gap between the nanoelectrodes. The AuNP devices have been characterized in the same manner as the nanowire devices. The thus determined conductance values are 12.8, 16.3, and 28.9 pS. These values are higher, by roughly a factor of ten, than the conductance values obtained for nanowire devices, but three orders of magnitude lower than conductance values obtained for single-AuNP devices, which are typically around 14 ± 3 nS, as reported in [15]. Representative conductance curves for the devices under consideration are given in Figure 3. Corresponding *I*/*U* curves in linear scale can be found in addition in Supporting Information File 1 (Figure S6). While Ru(MPTP)_2_-complex wire devices exhibit a clear linear dependence, *I* ~ *U*, multiple-Ru(MPTP)_2_–AuNP devices show a nonlinear current vs voltage behavior at voltages above 1 V pointing to a tunneling mechanism.

Besides the obviously different conductance values, no electrical switching could be observed for the Ru(TP)_2_-complex wire devices and the multiple-Ru(MPTP)_2_–AuNP devices in contrast to the single-Ru(MPTP)_2_–AuNP devices. Here it should be mentioned that all our attempts to enable electrical switching in multiple-Ru(MPTP)_2_–AuNP devices by increasing the applied voltage failed in our experimental voltage window of ±3 V, which is limited by the stability of the nanoelectrodes. For the nanowire devices a rather practical reason for the missing electrical switching might be the low conductance through these devices while the expected on/off ratio of 1.5 would lead only to a minor current increase [15]. Thus, the expected electrical switching effect in these devices may be simply covered by noise. However, to discuss this issue in more detail the conductance mechanism in the different devices needs to be clarified first. Additionally, it should be noted that quantum effects can be excluded in our AuNP-based devices, since the AuNPs exhibit sizes above 10 nm and the measurements are performed at RT and higher temperatures.

**Figure 4:**
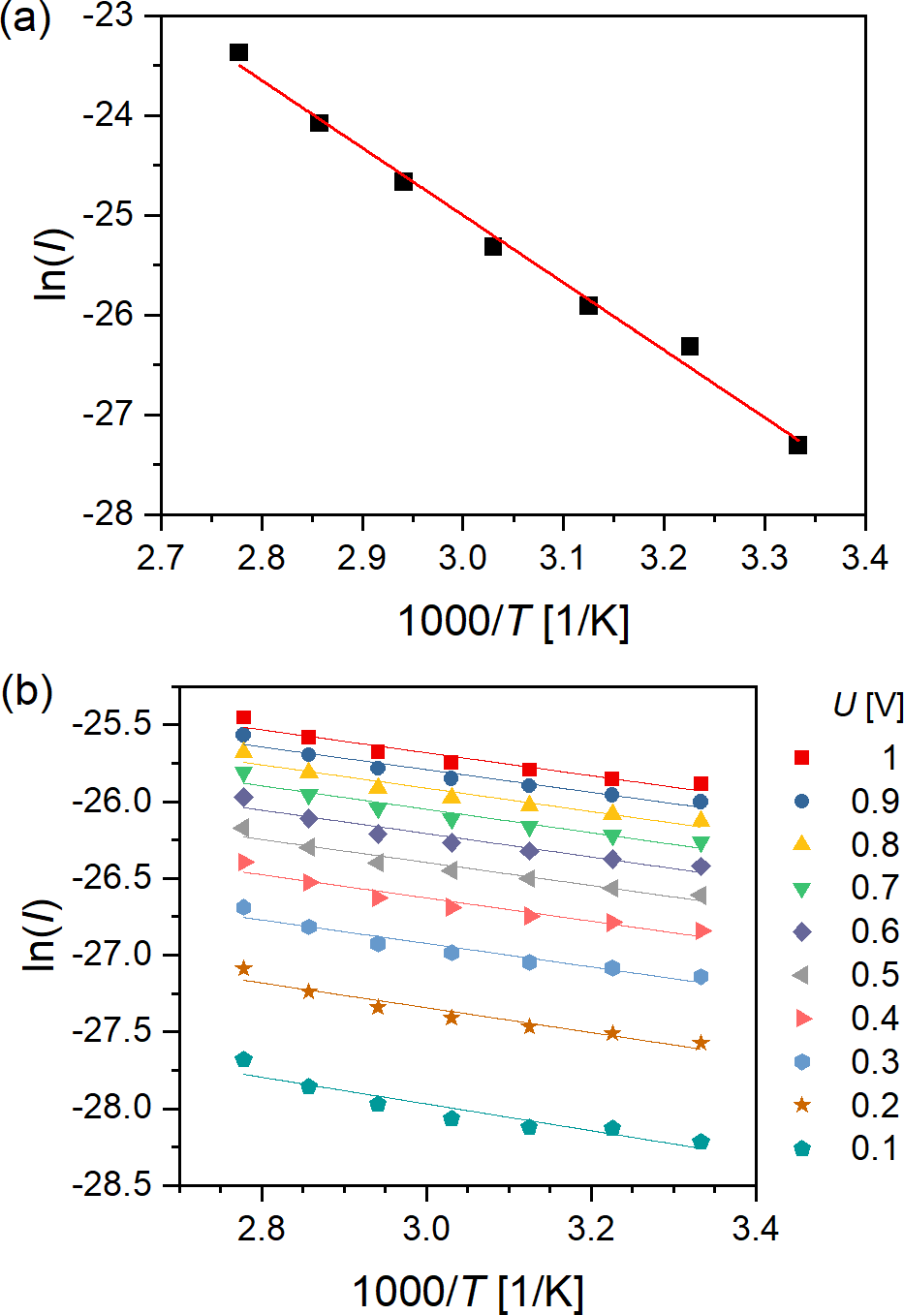
Arrhenius plots. (a) Ru(TP)_2_-complex wire device (*U* = 1 V). (b) Ru(MPTP)_2_–AuNP device for voltages between 0.1 and 1 V. Points correspond to measurements while lines correspond to linear regressions.

In order to determine the underlying conductance mechanism, temperature-dependent measurements have been carried out. For this, the current at a constant bias of 1 V was recorded at different temperatures in the range between 300 and 360 K. Figure 4a shows the resulting Arrhenius plot of a Ru(TP)_2_-complex wire device. In this case the linear regression reveals an activation energy of *E*_A_ = 582 meV. Over all eight samples, energies ranged from 367 to 584 meV with a median of 479 meV. Thus, it is reasonable to assume thermally activated hopping conduction for these solid-state devices, as it had been reported already for transition metal complex wires in solution [7,31–32]. According to literature, the most likely first step of the activated transport is the injection of electrons from the metallic cores (Ru^2+^) into the TP ligands, that is, the organic constituents (pyridine and phenyl groups) of the ligands. In subsequent steps, the electrons might hop from a pyridine (phenyl) group to another pyridine (phenyl) group or to oxidized metallic cores (Ru^3+^). Most interestingly, the lower limit of the activation energy determined for hopping conduction through the Ru(TP)_2_-complex wire device corresponds well to the energy offset, Δ*E*_H_ = *E*_F_ − *E*_HOMO_, between the Fermi energy of the contacting electrodes and the highest occupied molecular orbital (HOMO) of the Ru complex, which we have recently determined to 330 meV [15]. This suggests that Δ*E*_H_ is a relevant activation energy in ideal devices to be overcome for hopping conductance. Furthermore, *E*_A_ corresponding to Δ*E*_H_ points to hole conduction through the energetically accessible HOMO of Ru(TP)_2_-complexes, as also discussed in [31–32]. In practical nanowire devices other contributions such as an additional contact resistance or an activation energy for wire-to-wire hopping may become relevant, too. The absent length dependence, the linear correlation between device conductance and bias voltage, and the distinct temperature dependence of *E*_A_ point to a thermally activated hopping conduction in solid-state nanowire devices.

Temperature dependent *I*/*U* measurements on Ru(MPTP)_2_–AuNP devices are given in Figure 4b. Here, the current was measured for ten different biases, starting with 0.1 V and increasing in 0.1 V steps up to 1 V, at each temperature. The determined activation energies are considerably smaller than those deduced for Ru(TP)_2_-complex wire devices and decline with ascending voltage from *E*_A_ = 75 meV at 0.1 V to 64 meV at 1 V (see also Supporting Information File 1, Figure S7). This small decline in *E*_A_ can be attributed to the broadening of the electron energy distribution at the Fermi level, that is, the temperature-dependent Fermi–Dirac distribution, and indicates a tunneling mechanism [33–35]. A tunneling mechanism has been observed for single-Ru(MPTP)_2_–AuNP devices as well [15]. TEM images of Ru(MPTP)_2_–AuNP assemblies point to a nanoparticle separation of about 4 nm corresponding to slightly intercalating ligand shells or even the formation of dithiol bonds between adjacent ligand shells. Assuming a number of three Ru(MPTP)_2_–AuNP building blocks bridging the gap between the nanoelectrodes and applying the best practice to estimate the conductance for these AuNP devices a value of *G*_NP,theo_ = 25 pS results. This value is based on sequential tunneling from AuNP to AuNP, a separation of 4 nm between the AuNP, a decay constant of β = 3.1 nm^−1^ for the Ru(MPTP)_2_-complex (for details see Supporting Information File 1, section 8) and fits exemplarily the experimentally obtained conductance values. Hence, the decreased conductance of the few-Ru(MPTP)_2_–AuNP device can be directly related to the changed device geometry compared to single-AuNP devices, while the conductance mechanism remains tunneling transport. Thus, tunneling transport is determined for Ru-complexes bridging a gap of about 4 nm, that is, corresponding to the distance between two AuNPs, while in longer wires thermally activated hopping is the dominant transport mechanism. This finding is in notable agreement with reports from other groups [31–32,36]. Thus, the respective device design of Ru(TP)_2_-based devices with roughly comparable dimensions in the nanometer range determines the accessible conductance values (single-Ru(MPTP)_2_–AuNP ≫ few-Ru(MPTP)_2_–AuNP > Ru(TP)_2_ nanowire) and different conduction mechanisms. While single- and few-Ru(MPTP)_2_–AuNP devices exhibit sequential tunneling transport, Ru(TP)_2_-complex wire devices exhibit hopping transport, verified by temperature-dependent conductance measurements.

Next we discuss the impact of this finding on the electrical switching ability of few-Ru(MPTP)_2_–AuNP compared to single-Ru(MPTP)_2_–AuNP devices. The recently reported ideal single-AuNP devices are described as double-barrier tunnel junctions [15]. They are formed by two 2.2 nm tunneling gaps bridged by Ru(MPTP)_2_-complexes strongly coupled to the electrodes and to the AuNP. In this scenario, a transition voltage of 1.17 eV is needed to align the HOMO levels of the Ru complexes with the *E*_F_ of the electrodes, despite the considerably smaller energy offset between *E*_F_ and HOMO of Δ*E*_H_ = 0.33 eV. The relatively high transition voltage must be overcome to enable electrical switching of Ru^2+/3+^ in this device geometry. However, in a device consisting of three AuNPs, for example (Supporting Information File 1, section 8), in addition to single-AuNP devices two tunneling gaps of 4 nm must be considered as well as strong coupling between the Ru complexes and the AuNP but weak coupling otherwise. Assuming tunneling transport, the applied voltage will drop mainly over both 4 nm gaps leaving the HOMO levels of Ru(MPTP)_2_-complexes in the smaller tunneling gaps almost unaffected. Thus, only a part of the orbitals relevant for a continuous conductive path from one electrode to the other is shifted into the respective voltage window defined by the measurement conditions. This kind of voltage divider, determined by the few-Ru(MPTP)_2_–AuNP device geometry, in combination with the pinning of the Ru complex HOMO levels to the AuNPs impedes the formation of a higher conductive pathway between the electrodes, that is, a continuous path of electrically switched Ru^2+/3+^. An electrical switching of only a part of the relevant Ru complexes will have a minor effect on the device conductance if an on/off ratio = 1.5 is considered [15].

### Optical addressing

**Figure 5:**
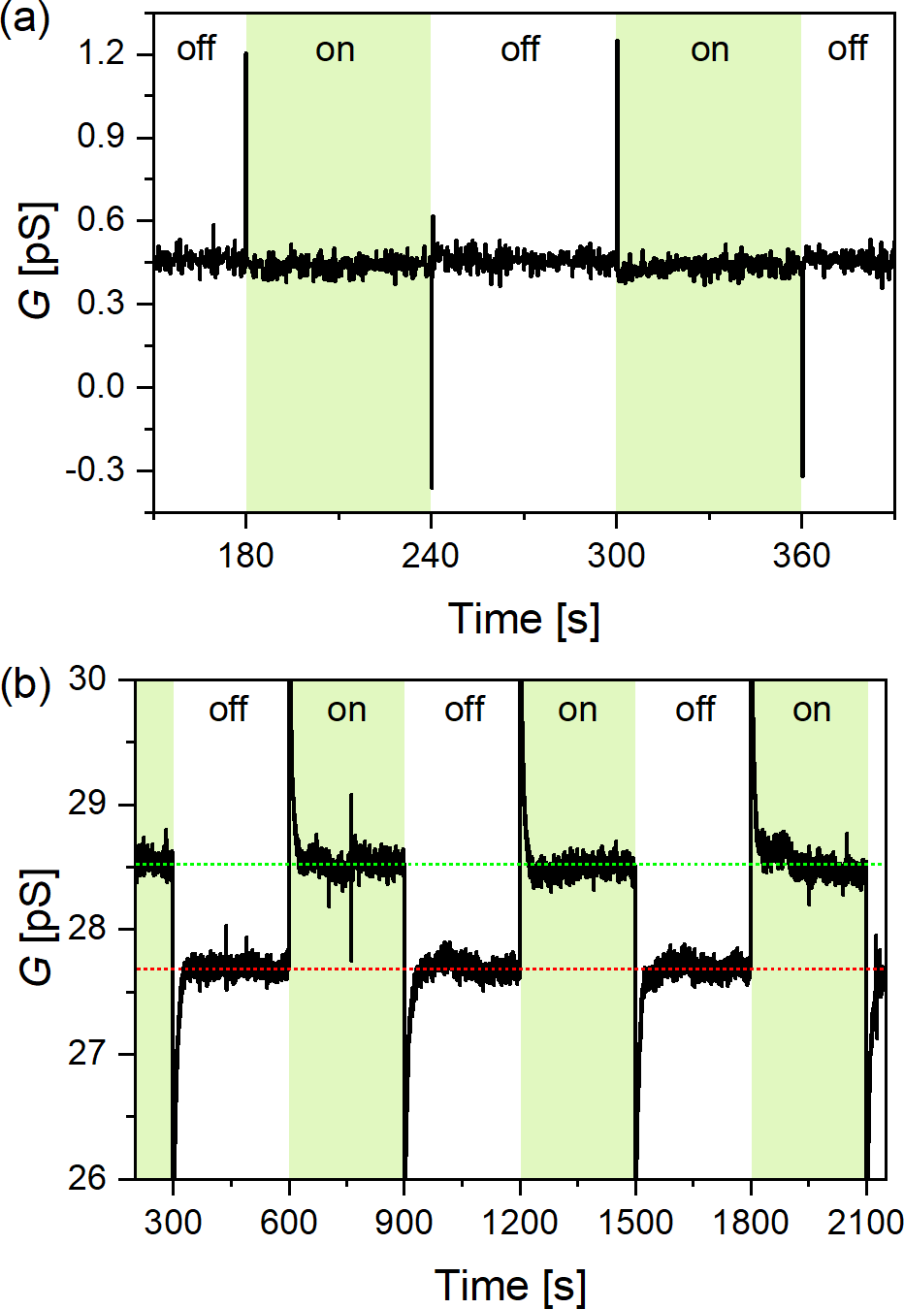
Current vs time traces at 1 V bias. Samples were illuminated with a 530 nm light. (a) Ru(TP)_2_-complex wire device. On/off durations of 60 s. (b) Ru(MPTP)_2_–AuNP device, with green and red dotted lines indicating the steady-state conduction values. On/off durations of 300 s.

Besides addressing the switching ability of Ru(TP)_2_-complexes by electrical means the influence of light illumination on these Ru(TP)_2_-complex devices is investigated. We studied the photoconductance properties of Ru(TP)_2_ devices upon irradiation at a wavelength of 490 to 540 nm and DC bias (*U*_SD_) of 0.1 to 1.2 V (Figure 5a). Current vs time traces were recorded while the light source was switched on and off for a period needed until a stable current was recorded. The period was varied between 30 s and several minutes. The time evolution of the current measured for Ru(TP)_2_ nanowire devices shows very short pulses corresponding to the light switching event. The time constant of these spike-like features could not be resolved in our measurement apparatus. However, they appear also in empty nanogap devices and are, therefore, attributed to plasmon excitations of the leads (see Supporting Information File 1, Figure S9). No significant difference in the steady-state current was observed for Ru(TP)_2_-complex wire devices under illumination and in the dark (on/off ratio given in Figure S10 of Supporting Information File 1).

The Ru(MPTP)_2_–AuNP devices, consisting of three to four AuNPs between the nanoelectrodes, were optically addressed by the same procedure, that is, 530 nm irradiation (*U*_SD_ = 1 V) and on/off switching with different frequencies and at bias voltages in the range from 0.1 to 1.1 V (Figure 5b and Supporting Information File 1, Figure S11 and Figure S12). Here it is possible, in contrast to Ru(TP)_2_-complex wire devices, to initiate conductance switching by applying an optical signal. We find a conductance ratio of 1.03 (at 1 V) between the steady-state current under irradiation (on) and in dark (off). This value is in line with data reported previously by us and described in literature for the switching ratio of Ru(TP)_2_-based monolayer devices [11,13,15].

**Figure 6:**
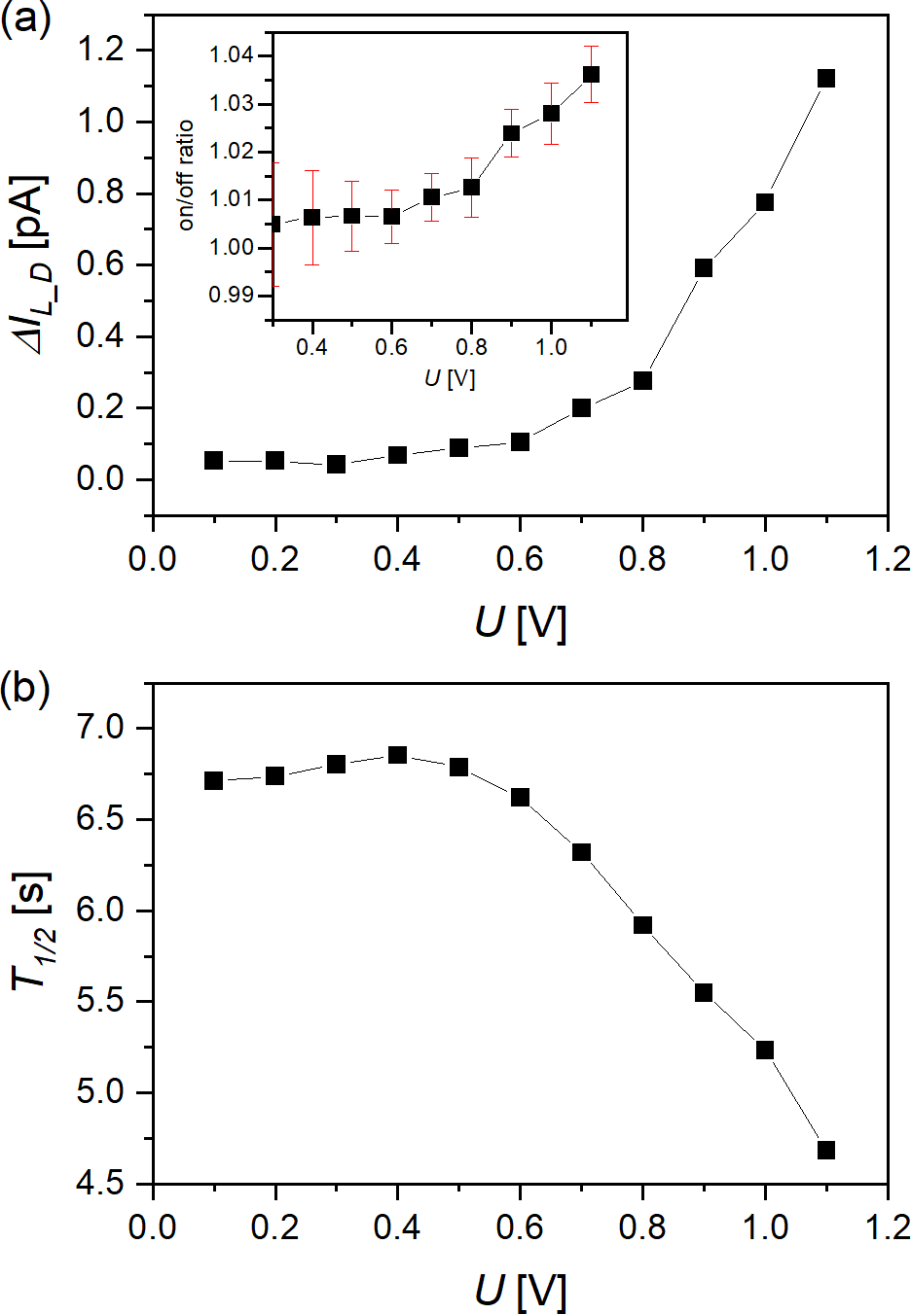
Photoconductance of Ru(MPTP)_2_–AuNP devices. (a) Difference between light current and dark current, Δ*I*_L_D_; inset: on/off ratio. Red bars indicate one standard deviation. (b) Half-life of current decline after the peak induced by switching the light source “on” or “off”.

Possible origins of this conductance switching in the Ru(MPTP)_2_–AuNP devices should be discussed. We can completely rule out a bolometric conductance enhancement like discussed for large nanoparticle arrays. We irradiate our device with 90 nW/cm^2^ only to exclude this influence, in contrast to otherwise used light intensities in the kW/cm^2^ range, which are described to cause an increase in temperature of approximately 0.55 K under certain conditions [37]. Furthermore, it is unlikely that the conductance switching in our Ru(MPTP)_2_–AuNP devices corresponds to photoconductance as discussed for AuNP arrays consisting of AuNPs coated with monolayers of organic molecules [38]. The photoconductance of these AuNP arrays is independent of *U*_SD_ and increases linearly with the intensity of the irradiating light, while they exhibit on/off ratios of less than 1.01 for light intensities of 1 µW/cm^2^. We find an increase of the photocurrent through the Ru(MPTP)_2_–AuNP devices due to irradiation above a threshold voltage of about 0.4 to 0.5 V (Figure 6a). Interestingly, this value corresponds well to the range of activation energies determined above for hopping transport in Ru(TP)_2_-complex wire devices.

In addition, we observe a prominent current peak in the current vs time traces of the Ru(MPTP)_2_–AuNP devices (Figure 5b) related to the switching on or switching off of the light source. This peak is not observed for Ru(TP)_2_-complex wire devices. Obviously, local surface plasmons are excited by the incident light and the oscillating electron density on the AuNP surface in the electrical field between the nanoelectrodes leads to a parasitic current, which is subsequently counterbalanced by charge carriers in the nanoelectrodes. Since surface plasmon excitations are in the femtosecond range and the diffusion of charge carriers is considerably slower, we find an exponential decay until a steady-state current is reached. The decay constant is field-dependent, as expected for the motion of charge carriers (Figure 6b).

The most likely explanation for the small current increase due to the illumination of Ru(MPTP)_2_–AuNP devices is that the AuNPs act as optical antennae, as described in [37–42]. This is supported by the increase of current due to irradiation with light of wavelengths larger than 520 nm corresponding to the surface plasmon band (see Supporting Information File 1, Figure S13), while no current increase is recorded at the wavelength corresponding to the MLCT band. The local surface plasmon resonance of the Ru(MPTP)_2_–AuNP is found at 533 nm, while the metal-to-ligand charge transfer band of Ru(MPTP)_2_-complexes is located at 499 nm. However, a considerable overlap of both bands is given (Supporting Information File 1, Figure S13) and, thus, a subsequent transfer of the incident electromagnetic energy to the Ru(MPTP)_2_-complexes bound to the AuNP surfaces is possible. Such a plasmon-induced resonance energy transfer is likely if a spectral overlap between the plasmon resonance and a molecular resonance, ideally of shorter wavelength, exists [41]. The transferred energy leads to the excitation of the redox center of the Ru(MPTP)_2_-complex, that is, a metal-to-ligand charge transfer is induced. The Ru^2+^ cation creates an exciton consisting of a threefold positive Ru^3+^ and a negative charge located on the MPTP ligand. Subsequently, these charge carriers are separated in the applied electrical field. Therefore, the electrical field needs a minimum energy of about 500 meV (Figure 6a), which is sufficient to initiate charge transport to the electrodes. Raising *U*_SD_ over this threshold voltage leads to a further increase in current. Consequently, we assume, that in Ru(MPTP)_2_–AuNP devices a plasmon-induced metal-to-ligand charge transfer is induced by irradiation with light of a wavelength of 530 nm, which finally leads to a small current increase through the device after charge separation of the excited [Ru^3+^(MPTP)_2_^−^]-complex in the applied electric field.

This supposed mechanism for current increase in Ru(MPTP)_2_–AuNP devices demands the adjustment of an effective equilibrium between light intensity, local surface plasmons of the AuNP, fraction of Ru(MPTP)_2_-complexes in the ground state, charge carrier density, density of trap states on the AuNP cores or TP ligands and the applied electric field. The resulting steady-state current depends on this equilibrium. The difference between the current under light illumination and the dark current, Δ*I*_L_D_, corresponds to the current enabled by plasmon-induced energy transfer to the Ru(MPTP)_2_-complexes and charge separation in an electrical field. This finding is in analogy to the plasmon-induced isomerization found in azobenzene derivatives used as ligands in nanoparticle arrays [41]. The conduction mechanism of the current contribution Δ*I*_L_D_ is assumed to be hopping conduction since the charge carriers are mainly located in the ligand shell of the AuNP. Furthermore, the activation energy for this process as well as the current range correspond well to the above described hopping transport through Ru(TP)_2_-complex wire devices. Thus, Δ*I*_L_D_ can only contribute a relatively small amount to the total current across the Ru(MPTP)_2_–AuNP device and consequently leads to a low on/off ratio.

Since it will be difficult to increase Δ*I*_L_D_, a straightforward way to improve the on/off ratio of Ru(MPTP)_2_–AuNP devices would be to increase the distance between adjacent AuNPs in the electrode gap, for example by employing longer ligand molecules. This will cause a decrease of the tunneling current contribution through the device, that is, an increase of the Δ*I*_L_D_ contribution to the total current, and thus an increase of the on/off ratio. These insights indicate that Ru(TP)_2_-complex wires are not so useful to bridge nanometer-sized gaps (<8 nm) between electrodes as single or few Ru(MPTP)_2_–AuNP building blocks in order to build small-area functional devices. This is caused by their extremely low conductance, the missing antennae effect of AuNPs in optical devices and, in consequence, the absence of relevant switching currents as response to an electrical or optical trigger. Moreover, the possibilities to tune Ru(TP)_2_–AuNP devices are more versatile.

## Conclusion

Overall, we demonstrated that hybrid materials from Ru(TP)_2_-complexes and AuNPs integrated in CMOS-compatible devices are useful switching elements that can be addressed by optical means. Furthermore, we could show that the device performance is sensibly determined by the molecule functionality and depends strongly upon the chosen device design, which can be used to define the transport mechanism. The use of long Ru(TP)_2_-complex wires bridging 8 to 20 nm gaps between nanoelectrodes results in hopping conduction with a low transport efficiency and the loss of significant switching ability. In a readily accessible approach using a few Ru(TP)_2_–AuNP building blocks with a diameter of 14 nm to bridge nanoelectrode gaps of 20 to 50 nm a light-driven conductance switching was measured. The underlying mechanism leading to an increase in current as response to light irradiation was identified as plasmon-induced metal-to-ligand charge transfer in Ru(TP)_2_ complexes and subsequent charge separation in the applied electrical field. Compared to Ru(TP)_2_-complex nanowire devices the performance of Ru(TP)_2_–AuNP devices is superior due to (I) the conductance mechanism based on sequential tunneling leading to a higher current, (II) the optical antennae effect of AuNPs, which allows for an energy transfer to the functional molecules, and (III) the ease and versatility of adopting the properties of the constituting elements according to the requirements of the respective devices. Our results reveal that the intrinsic properties of Ru(TP)_2_ complexes can be well preserved in Ru(TP)_2_–AuNP devices. We are convinced that this kind of devices based on functionalized AuNPs reveals the potential for the application in nanoelectronics or as sensors and we hope that our results inspire further developments in this field.

## Supporting Information

The Supporting Information includes the schemes of the chemical compounds used, SEM images of nanogaps and nanoparticles, XPS analysis performed during Ru(TP)_2_-complex wire growth, activation energies of Ru(MPTP)_2_–AuNP devices, a scheme for sequential tunneling in Ru(MPTP)_2_–AuNP devices, reference measurements and additional data of optically addressed devices.

File 1 Additional experimental data.
