# Supplementary material for: Impact of device design on the electronic and optoelectronic properties of integrated Ru-terpyridine complexes
Source: Beilstein J Nanotechnol. 2022 Feb 15;13:219–29. doi: 10.3762/bjnano.13.16 (PMC8895035; doi:10.3762/bjnano.13.16)
Supplement: File 1 — Additional experimental data. [file Beilstein_J_Nanotechnol-13-219-s001.pdf]

BEILSTEIN JOURNAL OF NANOTECHNOLOGY

Supporting Information

for

Impact of device design on the electronic and optoelectronic properties of integrated Ru-terpyridine complexes

Max Mennicken, Sophia Katharina Peter, Corinna Kaulen, Ulrich Simon and Silvia Karthäuser

Beilstein J. Nanotechnol. **2022,** *13,* 219–229. doi:10.3762/bjnano.13.16

Additional experimental data

License and Terms: This is a supporting information file under the terms of the Creative Commons Attribution License (https://creativecommons.org/licenses/by/4.0). Please note that the reuse, redistribution and reproduction in particular requires that the author(s) and source are credited and that individual graphics may be subject to special legal provisions.
The license is subject to the *Beilstein Journal of Nanotechnology* terms and conditions: (https://www.beilstein-journals.org/bjnano/terms)

1. Chemical compounds

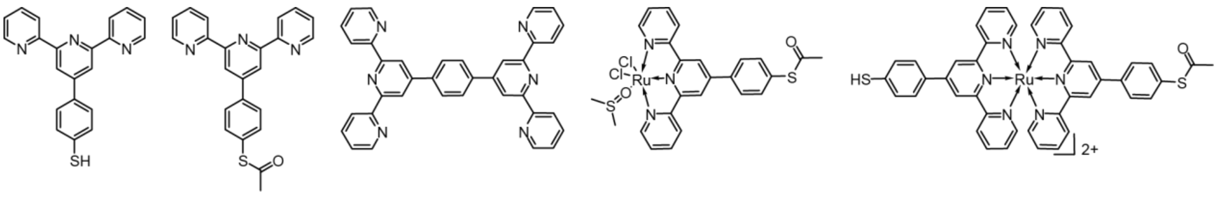

MPTP MPTP-SAc BTP $RuCl_2(DMSO)MPTP\text{-}SAc$ Ru(MPTP)(MPTP-SAc)

Figure S1: Chemical formulas of the substances used for synthesis: 4'-mercaptophenyl-2,2':6',2"-terpyridine (MPTP), 4'-[4-(acetylthio)phenyl]-2,2':6',2"-terpyridine (MPTP-SAc), 1,4-bis(2,2':6',2"-terpyridine-4-yl)benzene (BTP), as well as the Ru-complexes $RuCl_2(DMSO)MPTP\text{-}SAc$ and Ru(MPTP)(MPTP-SAc).

2. SEM image of $Ru(MPTP)_2$–AuNP

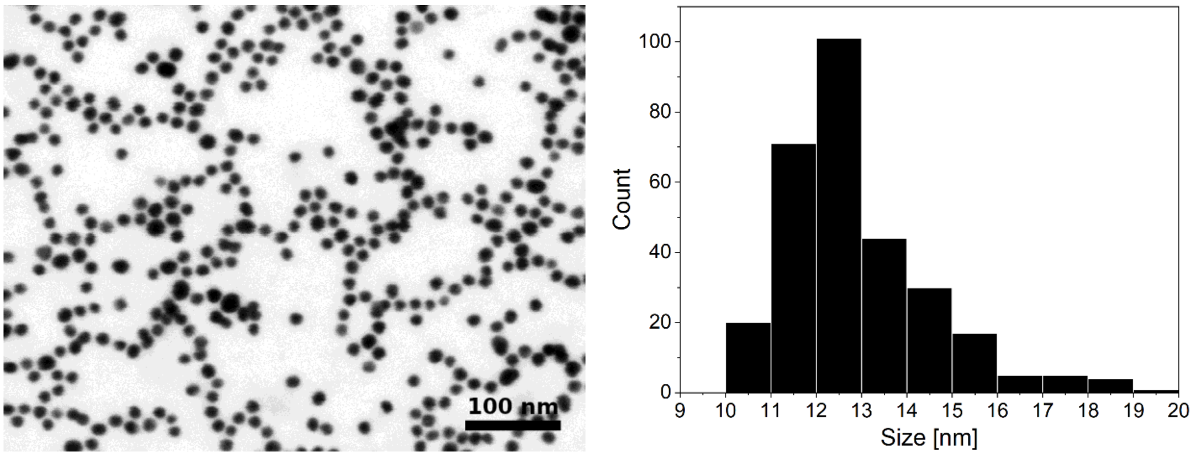

Figure S2: SEM image and corresponding histogram representing the size distribution of $Ru(MPTP)_2$–AuNPs. The deduced mean diameter is 12.9 ± 1.6 nm.

3. SEM images of nanogap devices

(a) 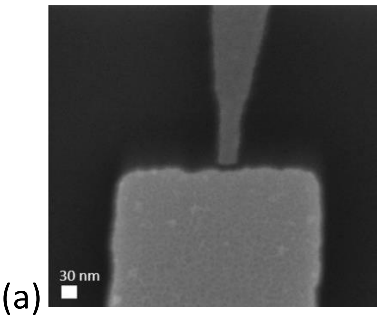

(b) 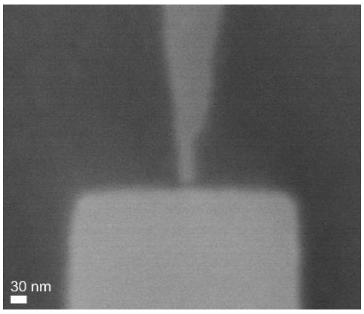

(c) 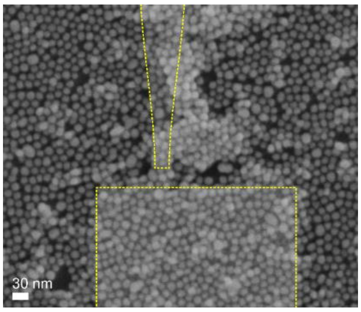

Figure S3: SEM images showing nanogap devices. (a) Empty nanogap device; (b) $Ru(TP)_2$-complex wire device; (c) $Ru(MPTP)_2$–AuNP device. Scale bars are 30 nm.

4. XPS measurements

XPS measurements have been performed during the first steps of the $Ru(TP)_2$-complex wire growth to support previously reported IRRAS and Raman spectra [1], since they can provide additional information due to their sensibility towards the oxidation state of ruthenium. Figure S4 shows the C 1s, Ru 3d and O 1s core level spectra. Since the C 1s and Ru 3d core levels, located around 285 eV and 282 eV, respectively, overlap to some extent, we followed the accepted procedure to deconvolute these peaks by fitting the ruthenium contributions first. The Ru 3d peaks are represented by a doublet ($3d_{5/2}$ and $3d_{3/2}$) with a separation of 4.17 eV and an area ratio of 3:2 [2]. As the Ru $3d_{5/2}$ peak is well resolved in most cases we will refer to this peak in the following.

Starting the wire growth with the chemisorption of MPTP on the Au substrate the core level spectrum in the range of 278 to 290 eV given in Figure S4a displays two C 1s BE. While the peak with low intensity at 286.7 eV (dashed blue line) corresponds to carbon directly bound to nitrogen, the main C 1s peak appears at 285.5 eV (solid blue line) in accordance with literature and contains contributions of all other carbons in MPTP, that is, the residual carbons in the pyridine rings and the phenyl ring (Table 1) [3]. In sample (ii), the main C 1s peak shifts by 0.5 eV to lower energies as a result of the first wire growth step, the complexation of MPTP with Ru-PF_6 in ethanol. This significant effect is caused by the fact that in surface sensitive methods like XPS the contribution of the near-surface cores to the total signal intensity is higher compared to low lying cores. In consequence, the main C 1s peak is shifted to a BE of 285.0 eV due to the contribution of the aliphatic carbons terminating the MPTP-Ru growth step while the contribution of aromatic and pyridine carbons is reduced [4,5]. The lower-intensity C 1s peak, observed at a BE of 286.2 eV, is assigned to the energy of carbon atoms involved in C–O bonds, like in ethanol [4]. These C 1s core levels of sample (ii) clearly indicate the presence of ethanol and suggest the formation of a $Ru(MPTP)(EO)_3$-complex (with EO corresponding to an ethoxide anion, $CH_3CH_2O^-$).

The corresponding Ru $3d_{5/2}$ peak at 282.1 eV, which can be identified as Ru(III) bound to hydroxide or in this case ethoxide ions, verifies this view (Figure S5b) [2]. In sample (iii), corresponding to MPTP-Ru-BTP wires, and sample (iv), corresponding to MPTP-Ru-BTP-Ru wires, no significant changes of the main C 1s peak can be observed. However, the low-intensity C 1s peak shifts alternatingly between 286.5 eV (C-N) and 286.2 eV (C-O) and strongly alternates in intensity, indicating a termination of the Ru-complex wire by either a TP group or a $Ru(TP)(EO)_3$-complex, respectively. A minor C 1s component observed in samples (iii) and (iv) at BE of 288.2 eV and 287.9 eV, is assigned to compounds containing C=O double bonds, like HCO_3^- species, which may result at Au surfaces due to air exposure [3-5].

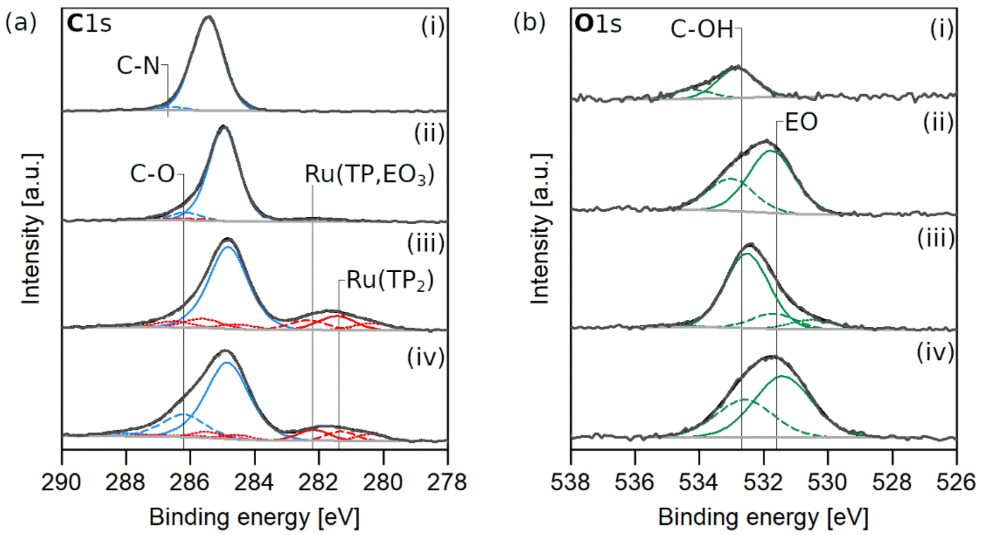

Figure S4: XPS of consecutive wire growth steps. Grey lines correspond to the actual signal while black lines correspond to the envelope of the blue and red fitting curves or the green fitting curves. (a) C 1s (blue) and Ru $3d_{5/2}$ (red) core level spectra, (b) O 1s core level spectra. (i) MPTP; (ii) MPTP-Ru; (iii) MPTP-Ru-BTP; (iv) MPTP-Ru-BTP-Ru.

The main Ru $3d_{5/2}$ peak observed during the Ru-complex wire growth from sample (ii) to (iv) tends towards a distinctly higher BE (282.1 eV), when the wire is terminated by the $Ru^{(III)}(TP)(EO)_3$-complex (samples (ii) and (iv)) compared to the BE (281.5 eV) attributed to $Ru^{(II)}(TP)_2$ complexes (sample (iii)) (Table 1) [2,3]. For the MPTP-Ru-BTP-Ru wire the contribution of both Ru complexes to the Ru 3d peak is nearly comparable due to the presence of both Ru complexes in the wire and the rate of yield of the wire growth steps less than 100% under these mild reaction conditions. An additional Ru $3d_{5/2}$ peak with lower intensity at a BE of 280.5 eV is obtained in step (iii). This peak corresponds to hydrated ruthenium oxide, like $Ru^{(IV)}O(OH)_2$ or $Ru^{(IV)}O(EO)_2$, and can be formed due to air exposure. This Ru $3d_{5/2}$ peak is also observed in the core level spectrum of sample (iv).

In addition, the O 1s peak assignment reveals the alternation of the groups terminating the Ru-complex wire, too. The O 1s core level spectrum of sample (i), MPTP chemisorbed on the Au substrate, shows

a residual amount of the solvent ethanol adsorbed on the surface indicated by the O 1s BE of 532.9 eV attributed to aliphatic C–O bonds and 534.2 eV corresponding to carbon-bound hydroxy groups involved in hydrogen bonds (Figure S5b and Table 1) [6,7]. However, the main O 1s peak in the spectra of sample (ii) and sample (iv) appears at BE of 531.8 eV and 531.4 eV, respectively, and indicates the wire termination by the Ru(TP)(EO)$_3$-complex as well as the C 1s and Ru 3d$_{5/2}$ spectra [6]. In contrast, mainly the signature of ethanol is found in the O 1s core level spectrum of sample (iii), whose termination can be compared with that of sample (i). Thus, the O 1s BE corresponding to aliphatic C–O bonds alternate with those indicating hydroxy or ethoxy groups and can be attributed to alternating terminations during the Ru(TP)$_2$-complex wire growth by TP groups and Ru(TP)(EO)$_3$-complexes. It should be mentioned that the remaining low-intensity O 1s peak at 530.8 eV in sample (iii) is assigned to hydrated ruthenium oxide and, thus, complements the low-intensity Ru 3d$_{5/2}$ peak [2]. Accordingly, the XP spectra presented here prove, in addition to earlier published results based on by other spectroscopic methods, that the desired wire growth can successfully be conducted [1].

5. Conductance statistics of Ru(TP)$_2$ devices

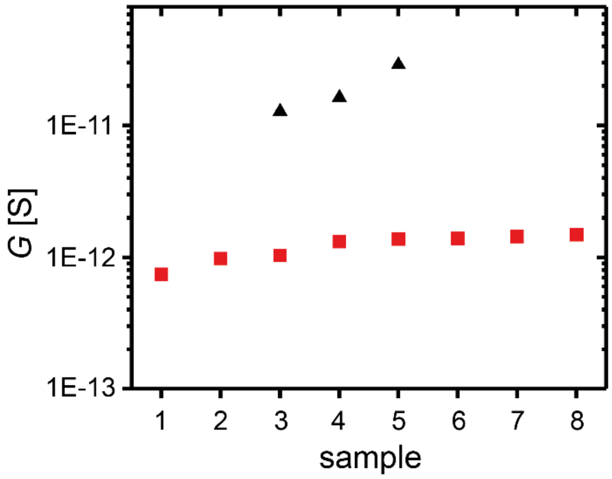

Figure S5: Experimental conductance statistics (at U_{SD} = 1 V) obtained from individual Ru(TP)$_2$-complex wire devices (red) and multiple-Ru(MPTP)$_2$–AuNP devices (black) on different nanoelectrode samples and measured under helium atmosphere (1 bar).

6. Linear current versus voltage curves

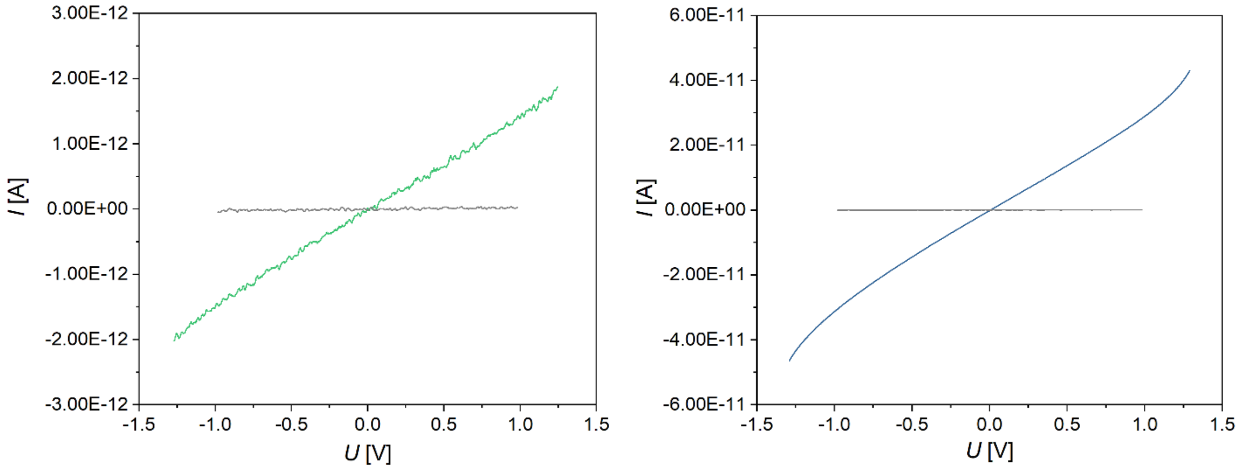

Figure S6: Linear current versus voltage curves of an exemplary Ru(TP)$_2$-complex wire device (left side, green) and a multiple-Ru(MPTP)$_2$–AuNP device (right side, blue) both including the I/U curve obtained from an empty nanogap representing the noise level (grey)). While Ru(TP)$_2$-complex wire devices exhibit a clear linear dependence, $I \sim U$, multiple-Ru(MPTP)$_2$–AuNP devices show a nonlinear current versus voltage behavior at high voltages pointing to a tunneling mechanism.

7. Activation energies of Ru(MPTP)$_2$–AuNP devices

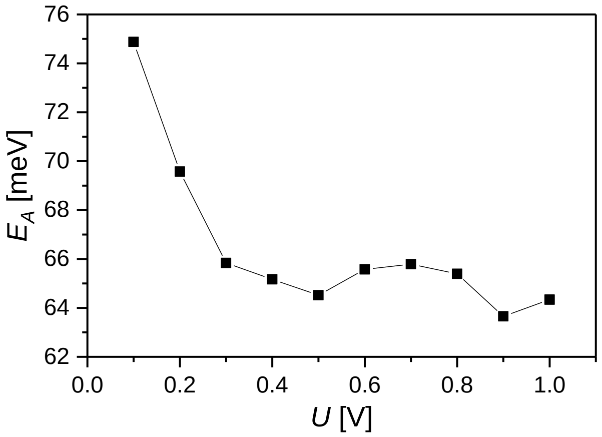

Figure S7: Activation energies, E_A, obtained from linear regressions of Arrhenius plots (ln(I) vs. 1/T) given in Figure 4. The curves, ln(I) vs 1/T, were measured for ten different biases in the range of 0.1 V to 1.0 V. A decline in E_A is determined for increasing bias and indicates a broadening of the electron energy distribution at E_F. This is in line with a tunneling mechanism.

8. Sequential tunneling through $Ru(MPTP)_2$–AuNP devices

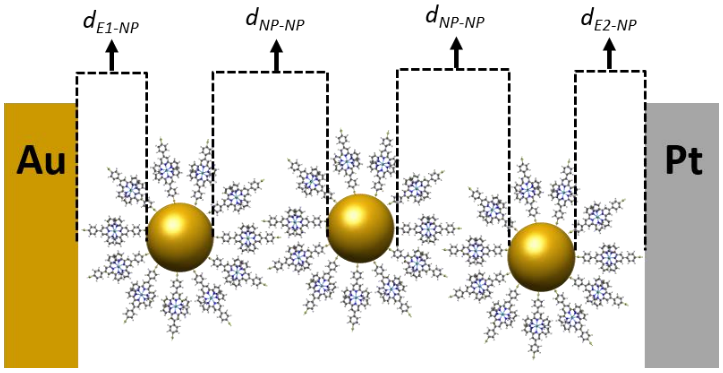

Figure S8: Schematic of three $Ru(MPTP)_2$–AuNP bridging the gap between heterogeneous nanoelectrodes with a separation of about 50 nm (not to scale).

We assume that transport in a $Ru(MPTP)_2$–AuNP device is based on tunneling of electrons through multiple small tunneling barriers, between an electrode and a AuNP or between two AuNPs, while a bias voltage is applied between the left and the right electrode. The tunneling barriers are formed by $Ru(MPTP)_2$-complexes. Considering the junction geometry given in the schematic, the device conductance (G_{dev}) is calculated using the series formula

$$\frac{1}{G_{NPdev}} = \frac{1}{G_{L-NP1}} + \frac{1}{G_{NP1-NP2}} + \frac{1}{G_{NP2-NP3}} + \frac{1}{G_{NP3-R}} \qquad (1)$$

with $G_{L\text{-}NP1}$ and $G_{NP3\text{-}R}$ the conductance values resulting from tunneling through the barrier between the left or the right electrode and the nearest AuNP, respectively, while $G_{NP\text{-}NP}$ corresponds to the conductance value through the tunneling barrier between two AuNPs.

The single-channel Landauer formula is applied to determine the theoretical device conductance at ±1 V according to the method we reported before [8-10]. In this model G through $Ru(MPTP)_2$-complexes forming the tunneling barrier between an electrode and AuNP or two AuNP is given by:

$$G = G_0\, T\, T\, e^{-ßd}. \qquad (2)$$

The values given in the following are used for the estimation of the device conductance: conductance quantum G_o = 77.5 µS, electronic decay constant for $Ru(MPTP)_2$ complexes $ß_{Ru}$ = 3.1 nm^{-1}, length of the $Ru(MPTP)_2$-complex $d_{Ru} = d_{E1\text{-}NP} = d_{NP\text{-}E2}$ = 2.2 nm, $d_{NP\text{-}NP}$ = 4.0 nm, transmission coefficients $T_{Au\text{-}SPhen}$ = 0.4 and $T_{Pt\text{-}SPhen}$ = 0.7 [8].

This procedure leads to a conductance value of G_{NPdev} = 25 pS for a $Ru(MPTP)_2$–AuNP device formed by three functionalized AuNPs between a nanoelectrode gap of about 50 nm and an interparticle distance of 4.0 nm as obtained by TEM analysis. This corresponds astonishing well to the median of the experimentally obtained values, G_{exp} = 16 pS, and confirms the above made assumption that sequential

tunneling through the Ru(MPTP)$_2$–AuNP device is the main transport mechanism. An evaluation of the uncertainties of this method to deduce the device conductance of AuNP devices, taking into account the error limits of the used parameter set, gap size uncertainties or even the influence of a possible vacuum gap and changes in device geometry, is found in [9].

9. Optical addressing of an empty nanogap

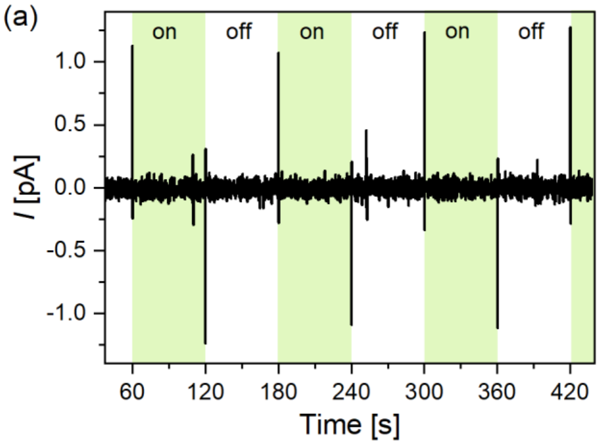

Figure S9: Current versus time traces obtained from an empty nanoelectrode gap of 30 nm at 1 V bias irradiated with 530 nm light at a frequency of 60 s. Obviously, spikes appear when the light is switched on or off. The same effect was obtained by interrupting the light beam with a sheet of paper manually.

10. On/off ratio of a Ru(TP)$_2$-complex wire device

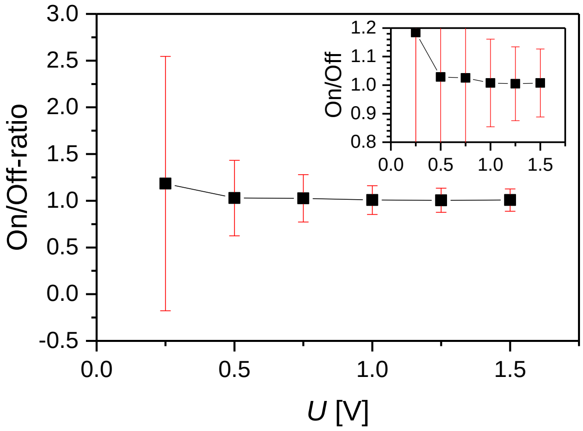

Figure S10: On/off conductance ratio determined between the steady-state currents of a Ru(TP)$_2$-complex wire device under irradiation and in the dark given for different bias voltages. Red arrow bars correspond to one standard deviation. The inset shows a zoom of the last five values. Due to the small conductance values of a Ru(TP)$_2$-complex wire device around 1 pS the standard deviation does not allow one to obtain on/off ratios below 1.1.

11. Optical addressing of $Ru(MPTP)_2$–AuNP devices

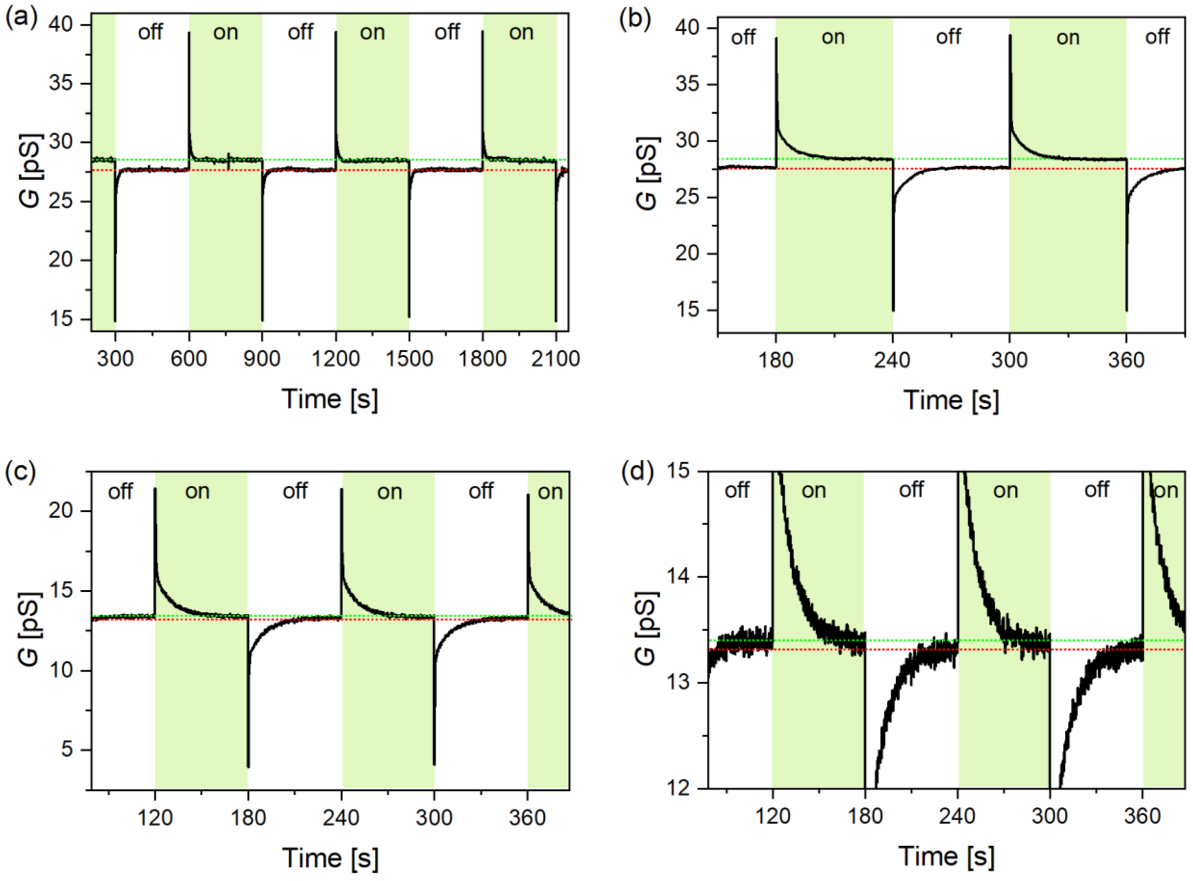

Figure S11: Current versus time traces from $Ru(MPTP)_2$–AuNP devices measured under different experimental conditions (wavelength, bias voltage, frequency). (a) 530 nm, 1 V, 300 s; (b) 530 nm, 1 V, 60 s; (c) 505 nm, 0.5 V, 60 s; (d) 505 nm, 0.5 V, 60 s.

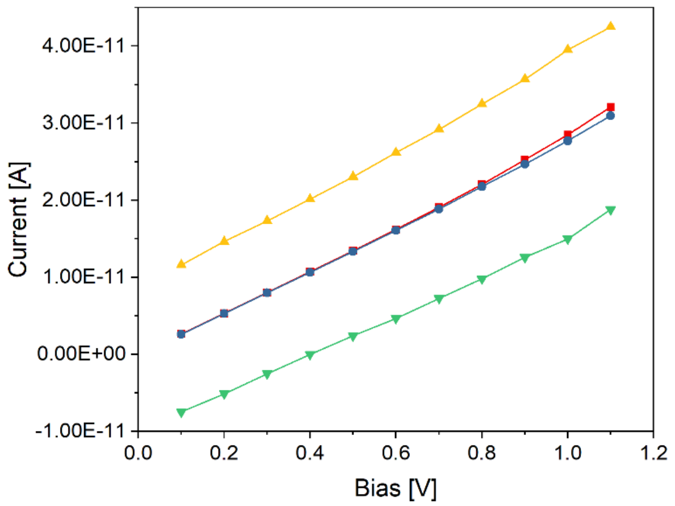

Figure S12: Current versus voltage diagram from a $Ru(MPTP)_2$–AuNP device showing the dependence of the steady-state current on the bias voltage, under illumination (red) and in the dark (blue). In addition, the bias voltage dependence of the peak currents is given (yellow and green), exhibiting an offset with respect to the steady-state currents (wavelength 530 nm, frequency 60 s).

12. Wavelength-dependent addressing of $Ru(MPTP)_2$–AuNP devices

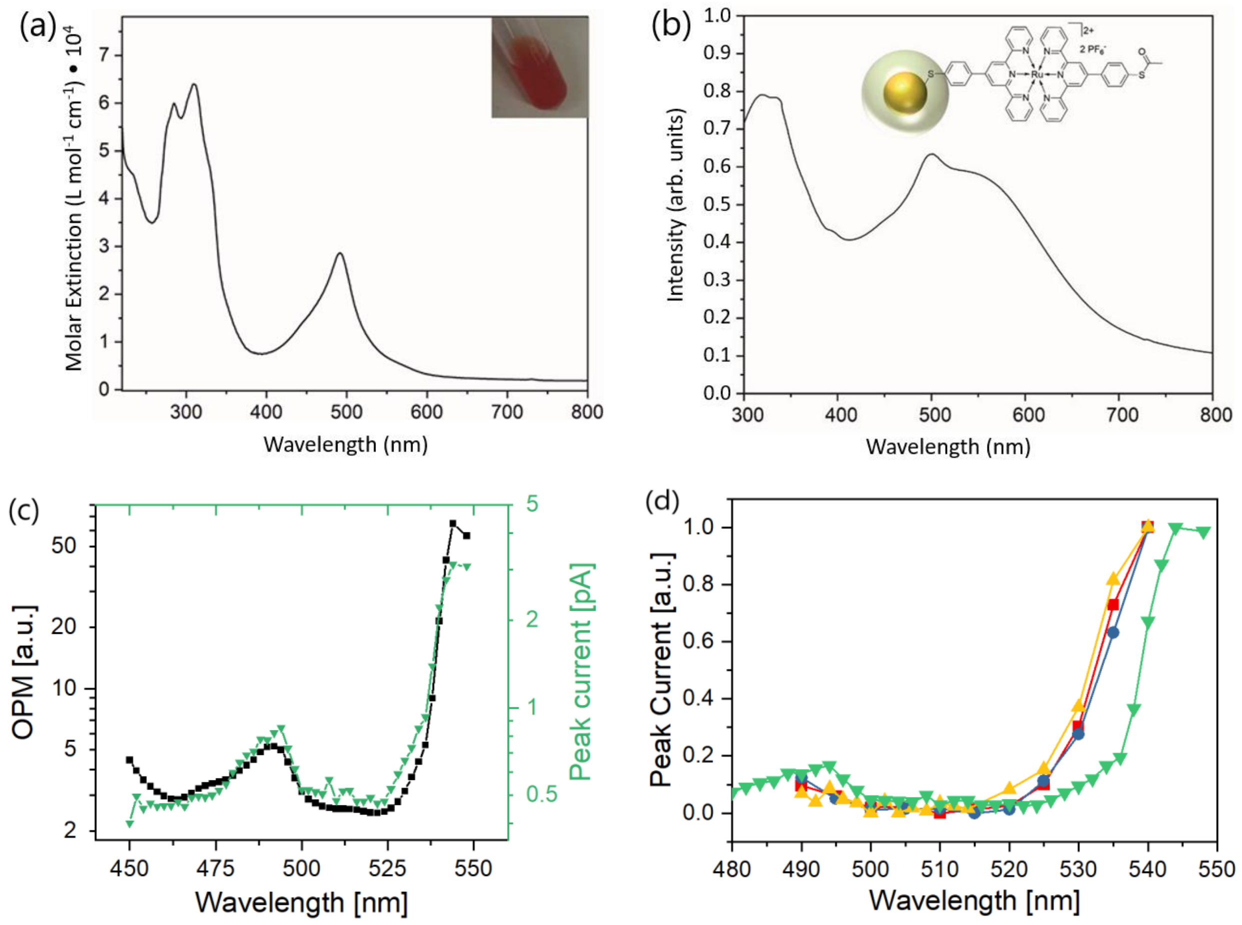

Figure S13: (a) UV–vis spectrum of Ru(MPTP)(MPTP-SAc)-complexes dissolved as 0.003 mM solution in acetonitrile exhibiting the MLCT band at λ = 493 nm. Inset: picture of a 1 mM Ru(MPTP)(MPTP-SAc)-complex solution (adapted from [11]). (b) UV–vis spectrum of Ru(MPTP)(MPTP-SAc)–AuNP showing the MLCT band of the $Ru(TP)_2$-complex at λ = 499 nm and the surface plasmon resonance of AuNP at λ = 533 nm (adapted from [11]). (c) Measurement of the peak current (maximum current of the spikes like shown in Figure S9 averaged over 3 spikes, at U = 1V) as a function of the wavelength used to illuminate an empty nanogap device (green). In parallel the OPM (optical power meter, black), that is the intensity of the irradiating light, was measured in steps of 2 nm. It is obvious, that the peak current is directly proportional to the intensity of the irradiating light. (d) Peak current of the three optically addressed multiple $Ru(MPTP)_2$–AuNP devices (yellow, red, blue; at U = 0.5 V) measured as a function of the wavelength of the irradiating light in the range of 490 to 540 nm with a step width of 5 nm. Additionally, the peak current for the empty nanogap (green) is given (same values as in (c)). For comparison of the wavelength-dependent current increase the current values of the different curves are normalized, that is, the maximum current is set to 1, while the minimum current is set to 0. Comparing the peak current curves in that way an increase in current is found for the multiple $Ru(MPTP)_2$–AuNP devices beginning at about 520 nm, while the current increase due to the wavelength dependent intensity of the Hg arc lamp starts

with a shift of about 10 nm to higher wavelengths. However, most interestingly, no increase in current is observed at 499 nm corresponding to the MLCT band of the $Ru(TP)_2$-complex. We conclude that the surface plasmon resonance of the AuNPs is addressed and that, subsequently, an energy transfer to the $Ru(TP)_2$-complexes takes place, which leads to an excitation of the redox center. Finally, the separation of the generated charge carriers in the applied electric field becomes manifested in the observed current increase.

References

1. Peter, S. K.; Kaulen, C.; Hoffmann, A.; Ogieglo, W.; Karthäuser, S.; Homberger, M.; Herres-Pawlis, S.; Simon, U. *J. Phys. Chem. C* **2019,** *123,* 6537–6548. doi:10.1021/acs.jpcc.8b12039
2. Morgan, D. J. *Surf. Interface Anal.* **2015,** *47,* 1072–1079. doi:10.1002/sia.5852
3. Agnès, C.; Arnault, J.-C.; Omnès, F.; Jousselme, B.; Billon, M.; Bidan, G.; Mailley, P. *Phys. Chem. Chem. Phys*. **2009**, *11*, 11647-11654. doi:10.1039/b912468c
4. Beamson, G.; Briggs, D. High Resolution XPS of Organic Polymers – The Scienta ESCA300 Database, Wiley Interscience, **1992**.
5. Naumkin, A. V.; Kraut-Vass, A.; Gaarenstroom, S. W.; Powell, C. J. (Eds.) NIST X-Ray Photoelectron Spectroscopy Database. Version 4.1, National Institute of Standards and Technology, Gaithersburg, **2012**.
6. Kerber, S. J.; Bruckner, J. J.; Wozniak, K.; Hardcastle, S. S.; Barr, T. L. *J. Vac. Sci. Technol., A* **1996,** *14,* 1314–1320. doi:10.1116/1.579947
7. Biesinger, M. C.; Lau, L. W. M.; Gerson, A. R.; Smart, R. S. C. *Appl. Surf. Sci.* **2010,** *257,* 887–898. doi:10.1016/j.apsusc.2010.07.086
8. Mennicken, M.; Peter, S. K.; Kaulen, C.; Simon, U.; Karthäuser, S. *J. Phys. Chem. C* **2020,** *124,* 4881–4889. doi:10.1021/acs.jpcc.9b11716
9. Babajani, N.; Kowalzik, P.; Waser, R.; Homberger, M.; Kaulen, C.; Simon, U.; Karthäuser, S. *J. Phys. Chem. C* **2013,** *117,* 22002–22009. doi:10.1021/jp405990y
10. Babajani, N.; Kaulen, C.; Homberger, M.; Mennicken, M.; Waser, R.; Simon, U.; Karthäuser, S. *J. Phys. Chem. C* **2014,** *118,* 27142–27149. doi:10.1021/jp5085179
11. Peter, S. K. Ruthenium Terpyridines on Gold Surfaces and Gold Nanoparticles for Applications in Nano-Electronics. Ph. D. Thesis, RWTH Aachen University, Germany 2020.
